# Feeding Problems and Their Underlying Mechanisms in the Esophageal Atresia–Tracheoesophageal Fistula Patient

**DOI:** 10.3389/fped.2017.00127

**Published:** 2017-05-31

**Authors:** Lisa Mahoney, Rachel Rosen

**Affiliations:** ^1^Aerodigestive Center, Division of Gastroenterology, Hepatology and Nutrition, Boston Children’s Hospital, Boston, MA, United States

**Keywords:** esophageal atresia, tracheoesophageal fistula, feeding difficulties, oropharyngeal dysphagia, impedance testing, aspiration, videofluoroscopic swallow study

## Abstract

Feeding difficulties such as dysphagia, coughing, choking, or vomiting during meals, slow eating, oral aversion, food refusal, and stressful mealtimes are common in children with repaired esophageal atresia (EA) and the reasons for this are often multifactorial. The aim of this review is to describe the possible underlying mechanisms contributing to feeding difficulties in patients with EA and approaches to management. Underlying mechanisms for these feeding difficulties include esophageal dysphagia, oropharyngeal dysphagia and aspiration, and aversions related to prolonged gastrostomy tube feeding. The initial diagnostic evaluation for feeding difficulties in a patient with EA may involve an esophagram, videofluoroscopic imaging or fiberoptic endoscopic evaluation during swallowing, upper endoscopy with biopsies, pH-impedance testing, and/or esophageal motility studies. The main goal of management is to reduce the factors contributing to feeding difficulties and may include reducing esophageal stasis, maximizing reflux therapies, treating underlying lung disease, dilating strictures, and altering feeding methods, routes, or schedules.

## Introduction

Children born with esophageal atresia (EA), with or without tracheoesophageal fistula (TEF), experience various gastrointestinal and respiratory complications and these complications often manifest with feeding difficulties; up to 75% of patients report difficulties with eating and the reasons for this are often multifactorial (1–7). Despite the high prevalence of these issues, the literature focused on feeding difficulties in these children is limited. While the focus of many studies is on esophageal abnormalities as the source of feeding difficulties, it is also important to consider oropharyngeal dysfunction and aerodigestive abnormalities as well (8). The aim of this review is to describe the nature of feeding difficulties in patients with EA, to discuss possible mechanisms for abnormal feeding, and highlight approaches to management in these patients.

## The Prevalence of Feeding Difficulties in Children with EA

A number of feeding problems have been described in children with EA, including dysphagia, liberal fluid intake during meals to help clear food boluses, coughing, choking, or vomiting during meals, slow eating, oral aversion, food refusal, and stressful mealtimes (9–11). In a study of 124 children with repaired EA, Puntis et al. characterized feeding difficulties and found that, compared to healthy controls, children with EA were significantly more likely to eat slowly, refuse meals, cough or choke during eating, and vomit with meals (9). In a recent review of 75 children (ages 0–16 years) seen in a multidisciplinary EA clinic, 79% had at least one problematic mealtime behavior with 54% of patients unable to consume age/developmentally appropriate textures, 29% with extremely selective eating behaviors, and 25% with lengthy mealtimes (10). While these feeding difficulties decreased with age, these rates are still extremely high (10, 11). While patients who have undergone primary repair of long-gap EA have delayed onset of feeding and significant variability in individual results, overall the major feeding milestones occurred in a similar pattern to normal infant controls (12).

Given the high prevalence of feeding difficulties in children with EA, providers should be aware of these issues and discuss feeding concerns with caregivers. Compared to normative sample controls, caregivers report significant feeding difficulties on validated feeding difficulty questionnaires: 17.5% of children with EA scored 1 SD above the mean and 6.7% scored 2 SDs above the mean (13). Even when present, the feeding difficulties were classified as mild in the majority of patients. Children with non-type C EA and those who were premature were more likely to have scores in the severe range (13). In a survey of 128 parents participating in an EA support group, 68% of parents reported that their children struggled with feeding difficulties including pain with eating, regurgitation of food, vomiting, burping, and avoidance of tough/bulky foods (14). Food impactions are also common in this population and 69% of parents reported that their child had at least 1 food impaction following their repair. Despite the widespread prevalence of feeding difficulties in patients with EA, few patients raise these concerns with their medical team; in a study by Puntis et al., only 11% of parents reported discussing feeding concerns during a medical visit (9). This suggests that targeted feeding questions should be included on all medical intake questionnaires and added to every follow-up clinical visit. While recognizing the problem is important, Ramsay and Birnbaum (15) took the recommendations a step further and recommended early involvement of a multidisciplinary team comprises occupational therapy, nutrition, and psychological support to assist families with feeding-related difficulties, and this recommendation has been supported by recent EA guidelines (16).

## Mechanism of Abnormal Feeding in Children with EA

### Esophageal Dysphagia

Esophageal dysphagia is common in patients with EA and causes include dysmotility, anatomic abnormalities, esophageal outlet obstruction, and esophagitis. While older children may present with complaints of food getting stuck, the presentation is often more challenging to discern in younger children. Symptoms in younger children include feeding difficulties, respiratory symptoms, vomiting, or poor growth (16). Dysphagia is present in 38–85% of patients with EA (1, 6, 7, 11, 17–19). Connor et al. found, in a systematic review and meta-analysis, an overall pooled estimated prevalence of 50.3% (3). Evaluation of dysphagia in a patient with EA may involve a number of diagnostic studies including (1) an upper GI contrast study assess for strictures or esophageal pooling, (2) videofluoroscopic swallow study (VFSS) to assess for aspiration and other causes of oropharyngeal dysphagia, (3) upper endoscopy to assess for esophagitis, and (4) esophageal motility testing to measure esophageal peristalsis and assess for bolus stasis if paired with impedance (8). Recent ESPGHAN–NASPGHAN guidelines recommend that all EA patients with dysphagia undergo at minimum an evaluation with an upper GI contrast study and esophagoscopy with biopsies for the evaluation of dysphagia, though in centers with motility capability, high-resolution esophageal manometry is helpful (16).

#### Esophogram

Barium imaging of the esophagus is helpful to identify esophageal strictures (congenital, peptic, or anastomotic), recurrent or missed fistulae, or pooling in the proximal esophageal pouch, all of which can contribute to feeding difficulties. Upper GI contrast studies are particularly helpful in patients with EA who have undergone fundoplication, where the fundoplication has the potential to create an esophageal outlet obstruction in the setting of esophageal dysmotility; in these patients, it is important to check delayed films to look for retained barium in the esophagus. Furthermore, following the barium into the stomach allows for imaging of slipped or herniated fundoplications. Holschneider et al. reported higher rates of postoperative dysphagia in children with EA who underwent fundoplication (17.2%) compared to those who underwent fundoplication for other indications (6.5%) (20). While there are no studies that directly address the role of fundoplication in feeding difficulties specifically in this population, patients with fundoplication can present with dysphagia, retching, volume intolerance during feeding, recurrent respiratory infections, and coughing after feeding, all of which have the potential to contribute to feeding difficulties.

#### Endoscopic Evaluation

Esophagitis is not uncommon in patients with EA and may be implicated as an underlying cause for dysphagia. In a study of 45 patients with EA undergoing upper endoscopy, Castilloux et al. found that although 31% of patients had histologic evidence of esophagitis, there was no association between symptoms of dysphagia and either gross or microscopic esophagitis (17). Sistonen et al. found similar results; while 25% of patients had esophagitis on histology, there was no relationship between inflammation and dysphagia (18). In another study by Deurloo et al., patients with dysphagia were more likely to have abnormal esophageal manometry studies, although there was no association between a reported symptom of dysphagia and a histologic diagnosis of esophagitis (21). While food impactions are often attributed to esophagitis, 38% of patients with EA who experienced food impactions actually had normal esophageal biopsies (17). This suggests that dysmotility may play a role in the pathogenesis of food impactions even in the absence of inflammation. All of these studies suggest that while treating esophagitis may be important, feeding issues are rarely a result of esophageal inflammation and setting realistic expectations for symptomatic improvement after acid suppression therapy for families is important.

Recently, there has been a growing body of literature on increased rates of eosinophilic esophagitis in children with EA. Dhaliwal et al. found a 17% incidence of eosinophilic esophagitis in a review of 103 patients with EA at a single center (22). This is higher than the incidence of eosinophilic esophagitis in the general population, which is estimated to be approximately 55/100,000 (23). Eosinophilic esophagitis should be a consideration in children with EA who have persistent symptoms despite appropriate antireflux therapy, progressive dysphagia, or recurrent strictures. However, because rates of recalcitrant reflux esophagitis may be higher in patients with EA because of the inability of a dysmotile esophagus to clear acid or because of inadequate acid suppression dosing, it is critical to determine if persistent esophagitis is incompletely treated reflux or eosinophilic esophagitis (16, 24–26). This evaluation may include additional testing with pH–MII to not only test for the amount of acid reflux but, if performed on therapy, also to assess for medication efficacy.

#### Multichannel Intraluminal Impedance with pH

While gastroesophageal reflux disease (GERD) is frequently reported in children with EA and objective diagnostic testing detects pathologic reflux in up to 67% of patients, recent literature suggests that feeding difficulties are not consistently associated with reflux events (24–29). In a study of 35 patients with EA who underwent pH–MII testing, Tong et al. found that only 19% of all dysphagia symptoms reported during pH–MII testing were associated with reflux events (24). Pedersen et al. studied 59 patients with EA and 25 controls who underwent pH–MII testing (26). Despite the fact that 70% of patients with EA reported dysphagia (compared to 20% of controls), there were no significant differences in any pH- or MII-parameters aside from the total number of acid reflux episodes that was actually higher in controls. In a study of 24 patients with repaired EA who underwent pH–MII testing, Fröhlich et al. found, using a standardized questionnaire, dysphagia with liquids in 13% of patients and dysphagia with solids in 58% of patients (28). However, there was no significant correlation between total symptom score based on questionnaire responses and either the reflux index (percentage of recording time with pH < 4) or the bolus index (percentage of recording time with esophageal exposure to a refluxate) on pH–MII testing. If pH–MII testing is considered in the evaluation of children with dysphagia or feeding difficulties, it must be analyzed not only by the software but also manually; baseline impedance values can be 75% lower than controls, so therefore software may underestimate reflux burden and symptom correlations (24, 28).

#### Esophageal Manometry

Low amplitude or absent esophageal peristalsis have been reported in many studies of esophageal motility in children with EA (18, 21, 30). In a study of 101 adult patients with EA, only 20% of patients had normal propagating peristalsis (18). Similar manometric findings were described by Deurloo et al.; 70% of subjects had low amplitude esophageal contractions and retrograde contractions were observed in 35% of subjects (21). Those patients who reported dysphagia were more likely to have abnormal esophageal motility along with significantly lower scores on health-related quality of life scales. For centers where manometry is not routinely performed, even radionucleotide esophagram studies reveal significantly longer esophageal transit times in patients with long-gap EA compared to those with non-long-gap EA, suggesting that imaging may be a potential adjunctive tool to help identify dysmotility (31). This suggests that distal esophageal dysmotility, rather than pooling over the anastomosis, may be a bigger contributor to feeding difficulties in many children and supports imaging to understand the pathophysiology of dysphagia first before dilation or other more aggressive interventions. From a prognostic perspective, there may be some improvement in esophageal peristalsis based on manometric studies as patients get older, although this needs additional validation using high-resolution manometry (32).

Standard manometry is limited because of the wide spacing between sensors that leave larger areas of the esophagus unmapped including areas of possible dysmotility and the lower esophageal sphincter. To overcome these limitations, high-resolution manometry catheters, which have up to 36 closely spaced sensors, allow for improved characterization of motility abnormalities in patients with EA. In a study of 40 children with repaired EA who underwent high-resolution manometry, Lemoine et al. found that 38% of patients had aperistalsis and 15% had evidence of pan esophageal pressurization (33). Both gastroesophageal reflux and pulmonary symptoms were more common in the aperistalsis group. However, it is critical to understand that symptoms often thought to be reflux-related are not, in fact, a result of increased numbers of reflux episodes but rather poor clearance of whatever reflux is present or retrograde movement of retained swallowed esophageal contents. Kawahara et al. reported absent mid-esophagus peristalsis in all 29 out of 29 patients studied with repaired EA; 17 out of these 29 patients also had absent contractions in the distal esophagus (30). This lack of peristalsis translates into poor bolus transit when compared to controls (28). Esophageal dysmotility may not be an entirely postoperative phenomenon and may not be unique to those with EA. Lemoine et al. reported esophageal dysmotility, with abnormal high-resolution manometry studies, preoperatively in two patients with isolated unrepaired tracheoesophagela fistula (34). These observations suggest that there may be abnormal development of the esophageal innervation and smooth muscle that contributes to the dysmotility seen in these patients.

### Oropharyngeal Dysphagia and Aspiration

One of the other contributors to feeding difficulties is oropharyngeal (rather than esophageal) dysphagia with resultant aspiration. Patients can present with food refusal, back arching, watery eyes, cyanotic spells, chronic respiratory infections, chest rattling, or noisy breathing before, during, or after feeding. The differential diagnosis for this oropharyngeal dysphagia includes laryngeal clefts, vocal cord paralysis or paresis, neuromuscular dyscoordination, or developmental delays in swallowing function. Hörmann et al. studied 25 VFSSs in 19 children with repaired EA (35). In this cohort, 16% of patients had nasopharyngeal regurgitation, 5% had had residue in the pharynx, 10% had laryngeal penetration, and 37% had aspiration. In a study of VFSS in 12 children with repaired EA, Coppens et al. found that 36% of patients had abnormal oral phases and 75% of children had abnormalities in the pharyngeal phase (36). Oropharyngeal dysphagia/aspiration is also an important factor in the long-term nutritional outcomes for children with EA; children who are at risk for aspiration are significantly more likely to be malnourished compared to children without aspiration that may be a combination of inadequate oral intake and increased metabolism related to recurrent respiratory infections and tachypnea (10). Once aspiration or penetration is diagnosed on VFSS, the following differential diagnoses should be considered to help predict prognosis.

#### Vocal Cord Paralysis or Paresis

Vocal cord paralysis is reported in 3–17% of patients with EA and may result from a combination of postoperative recurrent laryngeal nerve damage and prolonged or traumatic intubation (37–39). Morini et al. studied 174 patients with treated EA/TEF and found that 7 (4%) of patients had vocal cord paresis. Risk factors for vocal cord paresis in these patients included longer duration of time intubated, cervical esophagostomy, long-gap EA, and anastomotic leakage (37). Pediatric patients have high rates of recovery; in patients with vocal cord paralysis following cardiac surgery, for example, 35% of patients ultimately recovered vocal cord function with a median time to recovery of 6.6 months (40). The clinical implications are important because if vocal cord function is suspected to improve, placement of enteral feeding tubes may not be needed.

#### Laryngeal Cleft

Laryngeal clefts are included in the differential diagnosis of aspiration. In a recent study of children with EA undergoing rigid bronchoscopy and laryngoscopy, 26% of EA patients had a laryngeal cleft (41). In a case series of 183 pediatric patients diagnosed with laryngeal clefts, 22 (12%) patients had a TEF (39). Half of these patients presented with aspiration and 18% had feeding difficulties. Only 17 of the 22 patients with laryngeal clefts and TEF required surgical repair. Postoperative modified barium swallow studies showed resolution of aspiration following cleft repair (39). Again, the implications are important because if a laryngeal cleft can be repaired, enteral feeding tubes are not needed, and potential long-term feeding aversions can be avoided.

#### Neonatal Swallowing Dysfunction

The differential diagnosis for aspiration in a neonate includes neonatal swallowing dysfunction. Aspiration of thin liquids was observed in 68% of former preterm neonates referred for VFSS in a study of 148 patients done by Davis et al. (42). However, many of these patients eventually had improvements in their swallow function and ultimately went on to pass a repeat VFSS after a median of 3.4 months. While there are no studies assessing improvements in swallowing function over time in neonates with EA, the findings in the general neonatal population suggest that clinicians should consider repeating a swallow study to assess for improvement in swallowing before considering surgical interventions such as gastrostomy tube placement or fundoplication. Additionally, determining the natural history of this developmental condition in children with EA is critical to avoid unnecessary surgeries.

## Diagnosing Aspiration during Swallowing

Oropharyngeal dysphagia with resultant aspiration can be diagnosed by several different diagnostic tests. While there is no true gold standard test for aspiration, all testing modalities are considered complementary to one another. Studies comparing diagnostic testing modalities have found poor agreement between different studies. In a study of 63 children with cerebral palsy who underwent barium videofluoroscopy, salivagram, and milk scan for evaluation of aspiration, Baikie et al. found poor agreement between tests, with a maximum kappa of 0.20 (43). These results suggest that if aspiration is suspected, several different diagnostic modalities should be considered (8). The sensitivity of these tests in patients with EA is not known.

### Videofluoroscopic Swallow Study

The VFSS allows for visualization of the oral and pharyngeal phases of swallowing. Oropharyngeal aspiration diagnosed on VFSS is common in children. One large study of 300 symptomatic pediatric patients with feeding disorders undergoing VFSS found oropharyngeal aspiration in 34% of children (44). Of these patients, 81% had silent aspiration. Children with neurologic impairment (OR 4.65), developmental delays (OR 4.62), aspiration lung disease (OR 3.22), and enteral feeding (OR 2.03) were more likely to have silent aspiration. Weir et al. studied pneumonia risk in 150 children with swallowing dysfunction diagnosed on VFSS to determine if the results of VFSS predicted clinical outcome (45). On univariate analysis, the risk of pneumonia was significantly increased in patients with aspiration of thin liquids (OR 2.4) and in those with post-swallow residuals (OR 2.5), although there were no significant differences on multivariate analysis. Aspiration of consistencies other than thin liquids was not associated with any increased risk of pneumonia. However, the spectrum of pulmonary symptoms extends beyond just pneumonia and additional studies are needed to correlate findings on VFSS with other pulmonary manifestations. Another advantage of VFSS studies is that they can accurately identify primary, missed, or recurrent TEFs in addition to the primary swallowing dysfunction (46).

### Salivogram

In contrast to a VFSS that detects aspiration of a food bolus, aspiration of oral secretions can be detected using radionucleotide scintigraphy, and this may provide some insight into the severity of oropharyngeal dysphagia. In a study of 129 pediatric patients with suspected oropharyngeal dysphagia, Simons et al. found that aspiration was identified on 21% of studies (47). Factors associated with positive salivagram results included developmental delay (OR 2.8), chronic respiratory infections or pneumonia (OR 2.6), reactive airway disease exacerbations (OR 2.8), and use of H2 blockers or proton pump inhibitors (OR 2.7). Drubach et al. found a similar frequency of positive salivagrams (25%) in 222 children, with high agreement (kappa = 0.891, *P* < 0.0001) between salivagram and chest X-ray findings (48). In a study of developmentally normal children with recurrent lower respiratory tract infections, Somasundaram et al. found positive salivagrams in 39% of infants and 16% of children aged 1–2 years (49). There was no aspiration noted in children over the age of 2 years.

### Fiberoptic Endoscopic Evaluation of Swallowing (FEES)

Fiberoptic endoscopic evaluation of swallowing visualizes the pharynx and larynx during swallowing using a transnasal flexible fiberoptic laryngoscope, and this technique can be used to diagnose aspiration in both children and adults (50–54). FEES is the only study that can assess swallowing in infants while breastfeeding and is safe and effective in this population (55). Studies comparing FEES to VFSS have found low agreement between the two studies. In a study of 30 children undergoing both VFSS and FEES, da Silva et al. found low agreement overall between the two the studies, although laryngeal penetration and aspiration on FEES were associated with higher positive predictive value and specificity for abnormal VFSS (52). Kelly et al. studied 15 symptomatic adults who underwent simultaneous FEES and VFSS (51). Fifteen independent investigators from several sites reviewed the images and scored aspiration or laryngeal penetration. There was higher agreement between experts for the FEES images compared to VFSS. In a study of 126 adults with dysphagia, Aviv randomized participants to receive testing with either FEES or VFSS and monitored outcomes (54). Neither the incidence of pneumonia nor the pneumonia-free interval was significantly different between the two groups.

### High-Resolution Manometry

High-resolution manometry can also be used as part of the diagnostic approach to suspected aspiration. Omari et al. compared assessment of swallow function using high-resolution manometry with impedance (HRM-I) to VFSS in 20 adults with suspected aspiration and 10 healthy controls (56). The swallow risk index (SRI) was calculated from automated analysis of combined manometric and impedance variables. The authors found that the SRI could be used to predict aspiration on fluoroscopy. These findings suggest that measurements taken during HRM-I can be used in the diagnosis of aspiration and offer the benefit of no radiation. In a HRM-I study of 20 children with oropharyngeal dysphagia, higher SRI, elevated upper esophageal sphincter pressure, and longer impedance flow intervals predicted risk of aspiration on VFSS, suggesting this technology also holds promise for use in children (57). The added benefit of this technology in children with EA is that both the upper and lower esophagus can be simultaneously assessed to determine aspiration risk, the quality of peristalsis, and the degree of esophageal stasis, all of which can contribute to feeding difficulties.

### Cervical Auscultation

Cervical auscultation involves audible detection of breathing and swallowing sounds by using a microphone, stethoscope, or accelerometer placed over the neck. It offers an advantage over instrumental assessments of swallowing in that it is non-invasive and does not involve exposure to radiation. A recent randomized controlled trial studied the utility of cervical auscultation in children referred for suspicion of aspiration (58). Children were randomized to either a clinical feeding evaluation plus cervical auscultation group or to a clinical feeding evaluation only group. The ability to predict aspiration, using VFSS as a reference, was studied. The sensitivity for cervical auscultation plus clinical feeding evaluation was 85%, whereas the sensitivity for clinical feeding evaluation alone was 63%. The utility of this in children with EA is not known and may be complicated by the tracheomalacia sounds frequently heard in these children.

## Management of Oropharyngeal Dysphagia-Associated Feeding Difficulties

There are many causes for feeding difficulties, and the main goal of management is to reduce the factors contributing to these difficulties. This may include reducing esophageal stasis by dilating fundoplications, maximizing reflux therapies, treating underlying lung disease to improve cough, posttussive emesis and tachypnea (all of which can affect swallowing), dilating strictures, and switching formulas. Sometimes changing feeding schedules or adding cyproheptadine (both as an appetite stimulant and to improve gastric accommodation) improves oral intake by maximizing hunger, allowing for greater gastric volumes, and drying up oral secretions. Many causes of oropharyngeal dysphagia improve over time, and thus management decisions regarding feeding should be made in the context of the likelihood of improvement. Although there are no studies directly addressing the management of aspiration in children with EA, the available literature in other populations may offer useful insight into managing aspiration in these children.

### Thickened Oral or Gastric Feeds to Reduce Aspiration during Swallowing and Aspiration of Gastric Contents

Thickening may serve many roles including reducing aspiration during swallowing, reducing full column reflux, and reducing retching. From a reflux perspective, Wenzl et al. studied 14 healthy infants with reflux who underwent intraesophageal impedance measurement and pH monitoring while being fed alternating thickened feeds and standard formula (59). The frequency and amount of regurgitation were significantly lower when infants received the thickened formula. Horvath et al. found a similar improvement in regurgitation in a systematic review of 14 randomized controlled trials evaluating the efficacy of thickening for management of infant GER (60). However, as was seen in the Wenzl et al. study, thickening had no effect on the frequency of acid GER episodes, the number of reflux episodes lasting >5 min, or the reflux index. Given these results, thickening may serve an important role in the aspirating child when trying to prevent formula from entering the mouth.

A second benefit of thickening may relate to a direct impact on the stomach. Patients with gastrostomy tubes and fundoplications may have less retching and gagging with thickened feeds. In a study of 33 children, Pentiuk et al. found that more than half of patients studied had over a 75% reduction in retching and gagging when given a diet of pureed foods *via* gastrostomy tube (61). Similar findings were reported by Nishiwaki et al. in adult patients with percutaneous endoscopic gastrostomy tubes; patients who received a semisolid diet had a significantly lower percentage of GER when compared to those receiving a liquid diet (62). Differences in gastric emptying time did not appear to be a significant driver of these findings.

Finally, thickening helps with oropharyngeal dysphagia. Studies in both children and adults have shown that thicker liquids alter the temporal characteristics of swallowing, especially closure of the true vocal cords, and lengthen deglutition time (63, 64). In a retrospective study of 546 infants, children with silent aspiration had fewer acute respiratory infections requiring admission or emergency room visits when receiving thickened feeds than those without thickening (65). In a study of 15 infants with respiratory syncytial virus bronchiolitis, 9 were found to have abnormal VFSS studies (laryngeal or tracheal penetration or aspiration) with thin barium. However, repeat studies with thickened barium improved these abnormalities in all but one patient (66). In adult patients with neurogenic dysphagia, increasing bolus viscosity significantly improves the safety and efficacy of deglutition (67). While thickening improves swallow mechanics in many patients, its role in changing the timeline for full oral feeding or role as a caloric supplement to improve weight gain is not known.

### Bolus versus Continuous Feeds to Reduce the Risk of Aspiration of Gastric Contents

Clinicians often alter the type of feeding to try to reduce reflux burden and change the feeding interval to improve oral feeding. However, there is limited data to support this practice in pediatric patients, and most data come from studies in preterm infants. Corvaglia et al. (68) studied cardiorespiratory outcomes in 33 preterm infants who each received both bolus and continuous feedings *via* orogastric tube. The continuous feedings were associated with more total apneic periods, more apneic periods lasting >20 s and more hypoxic episodes when compared to bolus feedings. In a randomized trial of intermittent bolus or semicontinuous nasogastric tube feedings in 246 low birth weight preterm infants, Rövekamp-Abels et al. found significantly lower mean daily gastric residual volumes in the bolus group (69). However, gastroesophageal reflux, respiratory complications, and time to full oral feeds were not assessed as outcomes in this population. The impact of feeding type in EA patients is not known.

### Changing Feeding Schedule or Formula to Reduce Discomfort or Reflux That May Impair Oral Feeding

There is no data in children with EA though there is limited pediatric data in other populations. From a reflux perspective, while patients are conventionally told that small, more frequent meals are better in reducing reflux, there is no pediatric data to support this. In fact, the feeding frequency has more to do with the type of refluxate. Children fed more frequently have predominantly non-acid reflux, whereas a longer period of time after initiation of a feed is associated with more acid reflux events (70, 71).

While breastfed and formula-fed infants do not differ significantly with respect to reflux characteristics, the formula type may be important (70, 72). In a crossover study of 17 children with suspected GERD and cow’s milk allergy who underwent pH–MII testing while fed 24 h of amino acid-based formula and then 24 h of cow’s milk, the authors found a significantly higher total number of reflux episodes and also a significantly higher number of weakly acid episodes when infants were being fed the cow’s milk (72). Similar results have been reported in adults. Horiuchi et al. found more rapid gastric emptying and fewer episodes of aspiration in adults with gastrostomy tubes who were given an elemental diet versus a standard liquid diet (73).

Finally, there may be a role for significantly reducing gastrostomy tube feeds in order to stimulate hunger and wean from gastrostomy tube feeds. In a recent prospective randomized controlled study of children with gastrostomy tubes initially placed for feeding difficulties, those assigned to a hunger provocation program with reductions in tube feeding by 50% had significantly more success weaning entirely off tube feedings than controls who had reductions of only 20–25% (86 versus 9%, *P* < 0.001) (74). Despite the desire of families to have their children on oral feeding, there is a significant lack of resources to facilitate this transition. In a study by Gardiner et al., this lack of resources results in significant practice variation in transitioning patients to oral feeding, and this is a critical step for all infants including those with EA (75).

### Transpyloric Feeding

Transpyloric feeding may be helpful in some children with EA as it has the potential to help reduce reflux burden, reduce retching, and allows for safe nighttime feeds. Because the rates of reflux are similar in children who receive transpyloric feeding and those who had a fundoplication (76, 77), transpyloric feedings can be used as a fundoplication alternative until the feeding difficulties or reflux improve. This feeding method allows for infant and toddler growth without permanently obstructing the lower esophagus (with a fundoplication), which may be of great benefit in children with EA who have absent esophageal motility and are therefore at risk for stasis over the fundoplication. Transpyloric feedings have been shown to reduce risk of pneumonia in adults and children. Metheny et al. described significantly fewer pneumonias in a cohort of 428 critically ill adults when feeds were introduced distal to the second portion of the duodenum (78). Srivastava et al. compared outcomes in 366 children with neurologic impairment and GERD who underwent management with either fundoplication (323 children) or gastrojejunal tube feedings (43 children) (79). The authors found that overall survival and pneumonia-free survival was similar between the groups during the follow-up period (median 3.4 years).

### Fundoplication to Improve Feeding Tolerance

Fundoplications are commonly performed in children with EA, with reported rates between 39 and 59% of all patients with EA (5, 6, 17, 80). The recent ESPGHAN–NASPGHAN guidelines list refractory anastomotic stenosis, long-gap EA, poorly controlled GERD despite maximal medical therapy, long-term dependency on transpyloric feeding, and cyanotic spells as indications to consider antireflux surgery in children with EA (16). There are very few studies that address the role of fundoplication on feeding tolerance in patients with EA. In a recent study, Menzies et al. found that EA patients who underwent fundoplication had significantly poorer growth compared to those who did not have a fundoplication (10). This suggests that altering the anatomy with fundoplication may actually worsen dysphagia and volume tolerance into the stomach, contribute to feeding difficulties, and subsequently impair growth. Levin et al. found that EA patients who underwent fundoplication had higher rates of dysphagia postoperatively, compared to preoperative symptoms, regardless of surgical fundoplication technique (81). There were no significant differences in the rates of poor growth in the preoperative and postoperative settings in this cohort. Because of the relatively high rate of fundoplication in this population, additional studies on the impact on feeding are critical. Children with EA who are being considered for fundoplication should be evaluated with a barium contrast study, endoscopy with biopsies, and reflux testing preoperatively as well as esophageal motility testing whenever possible (16).

## Conclusion

Feeding difficulties are common in patients with repaired EA, and this review highlights possible underlying mechanisms for abnormal feeding. Esophageal dysphagia, due to esophageal dysmotility, musical inflammation, or anatomic abnormalities such as strictures, is well described in patients with EA. Oropharyngeal dysphagia with resultant aspiration can also contribute to feeding difficulties in these patients and can be under recognized as symptoms often mimic other conditions such as reflux. There are many diagnostic tests that can aid in diagnosis of dysphagia, and patients with EA often require multiple tests to arrive at the correct diagnosis. Management centers on reduction of underlying factors contributing to feeding difficulties while recognizing that many causes of esophageal and oropharyngeal dysphagia improve over time. Clinicians caring for patients with EA should have a high index of suspicion for feeding difficulties in their patients and management with a multidisciplinary team is recommended for optimal care.

## Author Contributions

LM and RR each contributed to the draft of the manuscript and approved the final draft submitted.

## Conflict of Interest Statement

The authors declare that the research was conducted in the absence of any commercial or financial relationships that could be construed as a potential conflict of interest. The reviewer JM and the handling Editor declared their shared affiliation, and the handling Editor states that the process nevertheless met the standards of a fair and objective review.

## Abbreviations

EA, esophageal atresia; VFSS, videofluoroscopic swallow study; TEF, tracheoesophageal fistula.
